# Small-Pox Developed in a Fœtus

**Published:** 1874-06-01

**Authors:** O. T. Maxson

**Affiliations:** Waukegan


### SMALL-POX DEVELOPED IN A FŒTUS.

EDITORS MEDICAL EXAMINER.—I have in my possession a fetus of about five month's uterine life, which, although not new to the profession, I think of interest, it being a well marked case, confirmatory of the absoluteness of protection of vaccine against small-pox.

Case, Mrs. F., a strong laboring woman, aged twenty-two years, became enciente about the first of November last. February 21st, her husband was attacked with fever of small-pox. She attended him until March 4th, when he died of confluent small-pox. She had previously been vaccinated, and was vaccinated again at this time, the last inoculation, however, did not take effect. She experienced no ill effects from her exposure, and never felt motion of her child. April 23rd, she called me to relieve an itching of her skin. So intense was it that she required constant rubbing by others for relief. Her skin was a bright scarlet; pulse 120, soft and compressible; tongue clear and moist; urine scanty; thinking some blood poisoning caused the disturbance I gave chlor. tinct ferri and spirits nit. dulc. About twelve hours later I was called again; this time was informed of her pregnancy and that she was in labour. Examination of her vagina showed labour too advanced to attempt interruption. She was delivered of a child well marked with small-pox pits, showing it had gone through the stage of suppuration, which had marked the face, body and limbs freely, before its death. She was pregnant four months when the fœtus contracted the disease; five months and twenty days old at its birth; weight, ten ounces; had been dead several days as the skin had commenced to macerate. The case is of interest in establishing the thoroughness of protection in vaccination and that the fœtus may pass through several stages of the disease without any unpleasant symptoms to the mother. The itching of the skin all passed off with the labour. She made a rapid recovery. I send the specimen to the museum of Chicago Medical College for the benefit of the profession.

O. T. MAXSON, M.D.

Waukegan, May 1, 1874.

